# On the Relationship between P3 Latency and Mental Ability as a Function of Increasing Demands in a Selective Attention Task

**DOI:** 10.3390/brainsci9020028

**Published:** 2019-01-29

**Authors:** Tugba Kapanci, Sarah Merks, Thomas H. Rammsayer, Stefan J. Troche

**Affiliations:** 1 Department of Psychology and Psychotherapy, University of Witten/Herdecke, 58448 Witten, Germany; tugba.kapanci@uni-wh.de (T.K.); 2 Institute Human in Complex Systems, School for Applied Psychology, University of Applied Sciences and Arts Northwestern Switzerland, 4600 Olten, Switzerland; sarah.merks@fhnw.ch (S.M.); 3 Institute for Psychology, University of Bern, 3012 Bern, Switzerland; thomas.rammsayer@psy.unibe.ch (T.H.R.)

**Keywords:** selective attention, mental ability, P3 latency, continuous performance test, mental speed

## Abstract

The mental speed approach to individual differences in mental ability (MA) is based on the assumption of higher speed of information processing in individuals with higher than those with lower MA. Empirical support of this assumption has been inconsistent when speed was measured by means of the P3 latency in the event-related potential (ERP). The present study investigated the association between MA and P3 latency as a function of task demands on selective attention. For this purpose, 20 men and 90 women performed on a standard continuous performance test (CPT1 condition) as well as on two further task conditions with lower (CPT0) and higher demands (CPT2) on selective attention. MA and P3 latency negatively correlated in the standard CPT, and this negative relationship even increased systematically from the CPT1 to the CPT2 condition but was absent in the CPT0 condition. The present results indicate that task demands on selective attention are decisive to observe the expected shorter P3 latency in individuals with higher compared to those with lower MA.

## 1. Introduction

Individuals with higher compared to those with lower mental ability (MA) have been reported to have shorter reaction times (RTs) in a wide range of elementary cognitive tasks (ECTs) [1,2,3]. ECTs are so easy that individuals with higher and lower MA do not differ in the number of errors or the use of cognitive strategies but only in speed of task completion. The most common explanation of the faster information processing in individuals with higher compared to those with lower MA refers to a more efficient information transmission in the central nervous system [4,5]. It should be noted that MA-related differences in mental speed can be observed in simple RT tasks but usually increase with increasing task demands [6,7,8]. Only after exceeding a certain level of task demands the relation between MA and RT decreases in favor of an increasing relation between MA and error rates in the experimental task [9,10].

To further elucidate the mechanisms underlying the relation between MA and speed of information processing, psychophysiological studies have probed whether MA-related speed differences can also be identified in the latencies of the event-related potential (ERP) [5,11]. ERP is an electrophysiological response to specific events or stimuli [12], which can be observed in an electroencephalogram (EEG). Different aspects of stimulus processing have been demonstrated to be related to the positive and negative components of ERP [13]. The P3 component, also referred to as P300 and first described by Sutton [14], is a very pronounced positive wave with a maximum peak at about 300 ms after presentation of a stimulus. If the stimulus is presented but not attended to, the P3 component does not (or only rudimentarily) emerge, indicating that the P3 component reflects the allocation of attentional resources [13,15,16,17]. More specifically, the P3 component is assumed to represent attention-related inhibition of ongoing brain activity to facilitate the consolidation of the target’s mental representation in working memory [15,16]. P3 latency, defined as the time interval between stimulus onset and the peak of the P3 wave, has been assumed to be a reliable index of the time needed to evaluate and categorize a presented stimulus [18,19,20,21,22]. As suggested by Verleger’s [23] thorough review, however, the view of P3 latency as a pure speed measure of cognitive processes unrelated to response processes might be premature, since P3 latency is also sensitive to delays in response selection when responses are given fast.

As an electrophysiological and reliable measure of speed of information processing [24], P3 latency also received much attention as a possible correlate of MA. In contrast to RT, however, P3 latency was found to be less consistently related to MA, with the majority of studies investigating young adults (but see Reference [25]). In simple and choice RT tasks, for example, a relation between P3 latency and MA could not be obtained [22,26,27]. Houlihan et al. [28] reported a positive relationship between MA and P3 latency in a short-term memory scanning task, whereas McGarry-Roberts et al. [22] reported MA to be negatively related to the P3 latencies derived from a short-term and a long-term memory task. The only task showing consistently the expected negative functional relation between P3 latency and MA was the oddball task with shorter P3 latencies in higher- compared to lower-MA individuals [25,29,30,31,32,33].

From the inconsistent results on the relation between MA and P3 latency, it can be concluded that P3 latency is not in general related to MA. Rather, this relation seems to depend on the respective task used to elicit the P3 component or, in other words, on the specific cognitive processes required by the given task. For example, P3 latency associated with simple or choice reaction time was consistently unrelated to MA [22,27,28], while studies on MA and P3 latency associated with short-term memory scanning produced inconsistent results [22,28]. To date, little empirical support is available for a functional relation between MA and P3 latency associated with long-term memory retrieval [22]. Only the information processing required by the oddball task led to a consistent relation between the associated P3 latency and MA. Thus, the oddball task represents a good starting point for a systematic investigation of the task conditions and, thus, the required cognitive processes necessary to yield faster P3 latencies in individuals with higher than those with lower MA. In the following, we outline why we expect that selective attention is the crucial cognitive process underlying the negative relation between P3 latency and MA.

The oddball task consists of a series of standard stimuli (e.g., the letter “O”) infrequently interrupted by the “oddball” (e.g., the letter “X”), to which participants respond. In other words, the task requires to direct attention selectively to an infrequently presented target and to respond with a key press. Given these task characteristics, the oddball task is reminiscent of the continuous performance test (CPT) [34]. With the CPT, the participants’ task is to monitor a stream of letters successively presented on a monitor screen and to press a designated key in response to a prespecified target letter (e.g. “X”). According to Riccio et al. [35], the CPT is one of the most popular clinical tasks to assess sustained attention and vigilance by means of RT and error scores. A most obvious difference between the oddball task and the CPT is that the distractors are always the same (frequent) stimulus in the oddball task, whereas different distractor stimuli are used in the CPT. Nevertheless, the attentional demands of both tasks (i.e., identifying a target among distractors for a given period of time) are highly similar.

Given these similarities between the CPT and the oddball task, the first assumption to be investigated in the present study was that the target-related P3 latency in the CPT is negatively associated with MA as suggested by the findings with the oddball task. In addition, we assumed that the selective-attention demands on the identification of a target among distractors are decisive for the relation between P3 latency and MA. To investigate this hypothesis, two further CPT conditions were applied in the present study. In a control condition, the process of selective identification will be eliminated by omitting distractor stimuli from the task and presenting only target stimuli. The absence of distractors should reduce the demands on selective attention. If these demands, in fact, account for the observed relation between MA and P3 latency, the negative association between MA and P3 latency would be expected to vanish in the control condition.

In the case that the process of selectively identifying a target among distractors is the decisive process underlying the relation between P3 latency and MA, this relation should become stronger with increasing task demands on selective attention. To test this hypothesis, in an attention-enhanced CPT condition, the demands on selective attention were experimentally increased. For this purpose, the stream of letters contained a regular as well as an italic ‘X’ as ‘invalid’ and ‘valid’ target letter, respectively. The italic ‘*X*’, but not the regular ‘X’, was defined as the valid target stimulus. Participants were instructed to identify and to respond to the valid target letter (*X*) but to ignore the invalid target letter (X) as a distractor. Thus, during the process of correctly identifying the valid target stimulus, the letter as well as the font type needed to be attended to. If selective-attention demands for identifying targets among distractors represent the crucial source underlying the functional relationship between P3 latency and MA, the association between MA and the target-related P3 latency should increase in this latter condition compared to the standard CPT condition. With this approach, the present study aims to elucidate the necessary preconditions for a negative relationship between MA and P3 latency to occur. Learning more about these preconditions will contribute to a better understanding and conceptual expansion of the mental-speed approach to MA.

## 2. Materials and Methods

### 2.1. Participants

The sample consisted of 116 German-speaking undergraduate students. Due to extremely long RTs in one of the three CPT conditions (four participants) or implausibly low scores in the intelligence test (two participants), six participants were discarded from further analysis. The remaining 90 female and 20 male participants ranged in age from 18 to 36 years (mean = 22.0 years; standard deviation = 3.1 years). They reported normal hearing and normal or corrected-to-normal vision. Only healthy participants were tested and asked to refrain from caffeine and nicotine intake 2 h and from consuming alcohol at least 24 h prior to the EEG recording. As compensation for their participation, participants received course credit. Prior to testing, all participants gave their written informed consent. The study was conducted in accordance with the Declaration of Helsinki and the study protocol was approved by the ethics committee of the Faculty of Human Sciences of the University of Bern (Bern, Switzerland) (date of approval: 26 August 2013; project identification code: No. 2013-8-504570).

### 2.2. Assessment of Psychometric Intelligence

As a measure of MA, the German version of Cattell’s Culture Fair Test-20 R (CFT-20 R) [36] was used. It consisted of three subtests with 27 items (series, classifications, and matrices) and one subtest with 20 items (topologies). Weiss [36] reported test–retest reliabilities ranging from *r*_tt_ = 0.80 to *r*_tt_ = 0.90. Test-taking time was about 60 minutes.

### 2.3. Continuous Performance Test (CPT)

*Apparatus and stimuli*. All stimuli were presented on a 17’’ Dell computer monitor. Stimulus presentation was controlled by E-prime 2.0 experimental software (Psychology Software Tools, Inc., Sharpsburg, PA, USA). Stimuli were the letter X (target stimulus) and the letters G, D, A, W, M, S, K, and R (distractors) presented with a height of 1 cm and a width of 0.5 cm. All stimuli were presented in white font (Courier New, size: 28) against a black background. Participants’ responses were recorded by a Cedrus® response pad (RB-830) (Cedrus Corporation, San Pedro, CA, USA) with an accuracy of ± 1 ms. 

*Procedure*. Individual experimental testing and EEG recording took place in a sound-attenuated and electrically shielded room. Participants were seated in front of the computer monitor with a distance of 50 cm ensured by a chin rest. 

The experimental task consisted of three conditions (CPT0, CPT1, and CPT2). In all three conditions, a trial started with the presentation of a stimulus for 200 ms, followed by a black screen for 1000 ms. The next trial started after an intertrial interval randomly varying between 0 ms and 1000 ms.

In the 120 trials of the standard CPT condition (henceforth CPT1), the target stimulus (‘X’) was presented in 24 trials, and each of the eight distractors was presented in 12 trials. Participants were required to respond only to the target by pressing a designated response key as fast as possible (but to avoid errors). The order of trials was randomized. The second condition (CPT0), serving as a control condition, consisted of 32 trials. In each trial, the letter ‘X’ was presented. Participants were instructed to respond to the onset of the ‘X’ as fast as possible (but to avoid errors) by pressing the response key. Responses were recorded during the 1200-ms duration of a trial. The attention-enhanced condition (CPT2) was composed of 240 trials. The target stimulus was the letter ‘*X*’ (in italic font) presented in 24 trials. In addition to the abovementioned letters, which were presented in 192 trials, the letter ‘X’ (non-italic font) served as distractor in 24 trials. Participants were requested to respond to the ‘*X*’ in italic font as fast as possible but neither to the distractors nor to the ‘X’ in non-italic font. The duration of the CPT0 condition was about 1‘15 min; the durations of the CPT1 and CPT2 conditions were 4’30 and 8’45 min, respectively. 

Prior to the task, a general instruction was given and specific instructions; practice trials also preceded each task condition. The order of the three conditions was counter-balanced across participants. The total time to perform the CPT was about 15 to 20 min. As dependent variables, mean RT and error rates in each condition for each participant were determined. Mean RT was based on correct trials with RTs between 100 ms and 1200 ms. Error rates were analyzed separately for errors of omission (failure to respond to a target stimulus) and commission (responding erroneously to a distractor stimulus). 

*Electrophysiological recording*. During the CPT, EEG was recorded using a BrainAmp® amplifier (Brain Products GmbH, Gilching, Germany) and an electrode cap (EasyCap GmbH, Woerthsee-Etterschlag, Germany), with 12 Ag/AgCl electrodes referenced to the ear lobes. To control vertical and horizontal eye movements, electrodes were fixed below and above the right eye (vertical electrooculogram) and at the temples (horizontal electrooculogram). Impedances were kept below 5 kΩ. The sampling rate for the EEG signal was 1000 Hz, and the resolution was 0.1 μV. For further analyses, BrainVision Analyzer 2 (Brain Products GmbH, Gilching, Germany) was used. The EEG data were high-pass (0.1 Hz) and low-pass filtered (30 Hz). Eye movements were corrected by the regression-based method as proposed by Gratton, Coles, and Donchin [37]. The EEG was segmented based on the markers from the CPT sent with every onset of a stimulus. The duration of each segment was 1000 ms, with a 100-ms pre-stimulus and a 900-ms post-stimulus interval. Using a semiautomatic artifact rejection, segments with a voltage change above 500 μV within 1 ms, voltage changes above 200 μV within 200 ms, and values above 100 μV and under −100 μV were marked and rejected semiautomatically. A baseline correction for the pre-stimulus interval was done for each segment. Finally, the segments referring to the targets of each condition were averaged for each participant. A semiautomatic peak detection helped to find out the largest positive deflection for each individual within a time interval ranging from 190 to 550 ms after stimulus onset. If necessary, the peak was manually adjusted. This peak was considered as P3 amplitude. We focused on the PZ electrode site where the P3 component had the largest deflection in all three task conditions. The time between stimulus onset and this peak was defined as P3 latency. 

The complete experimental session also included an additional Hick RT task not relevant for the present study. Half the sample worked on the CPT prior to the other task, while the order was reversed for the other half. The results of the present study were not influenced by the order of task presentation.

## 3. Results

### 3.1. Behavioral Data

The mean CFT-20 R score (± standard deviation) was 77.1 ± 7.8, which is equivalent to a transformed mean intelligence quotient (IQ) of 98.8 (± 11.4). Table 1 provides means and standard deviations of RT in the three conditions of the CPT. A one-way analysis of variance (ANOVA) with CPT conditions as three levels of a repeated-measures factor revealed statistically significant differences in RT, *F*(2, 218) = 1978.510, *p* < 0.001, *η_p_*² = 0.948. As indicated by Bonferroni-adjusted post-hoc *t* tests, RT increased significantly from the CPT0 to the CPT1 condition, *t*(109) = 46.700, *p* < 0.001, *d* = 4.453, as well as from the CPT1 to the CPT2 condition, *t*(109) = 17.869, *p* < 0.001, *d* = 1.704. 

Also given in Table 1 are descriptive statistics of errors of omission in the three CPT conditions and errors of commission in the CPT1 and CPT2 condition. (Due to the lack of distractors in the CPT0 condition, participants could not make errors of commission in this condition.) As indicated by a one-way ANOVA on omissions with the three CPT conditions as levels of a repeated-measures factor, omissions differed significantly between the three task conditions, *F*(2, 218) = 11.550, *p* < 0.001, *η_p_*² = 0.096. There were significantly more errors of omission in the CPT0 than in the CPT1 condition, *t*(109) = 4.762, *p* < 0.001, *d* = 0.454, while omissions in the CPT1 and the CPT2 did not differ significantly after Bonferroni adjustment, *t*(109) = 2.152, *p* = 0.034, *d* = 0.205. Errors of commission, however, were made more frequently in the CPT2 than in the CPT1 condition, *t*(109) = 11.317, *p* < 0.001, *d* = 1.079.

Pearson correlations between RT in the three conditions are reported in Table 2. All three correlation coefficients yielded statistical significance. Unexpectedly, however, the correlations between RTs in the three CPT conditions and CFT-20 R scores were not significant and even positive in the most demanding task condition. 

Similarly, CFT-20 R scores did not significantly correlate with errors of omission in the three task conditions (CPT0: *r* = −0.006, *p* = 0.949; CPT1: *r* = −0.132, *p* = 0.171; CPT2: *r* = −0.101, *p* = 0.293) nor with errors of commission in the CPT1, *r* = −0.054, *p* = 0.578, and in the CPT2 condition, *r* = −0.116, *p* = 0.229.

### 3.2. Electrophysiological Data

**Figure 1:**
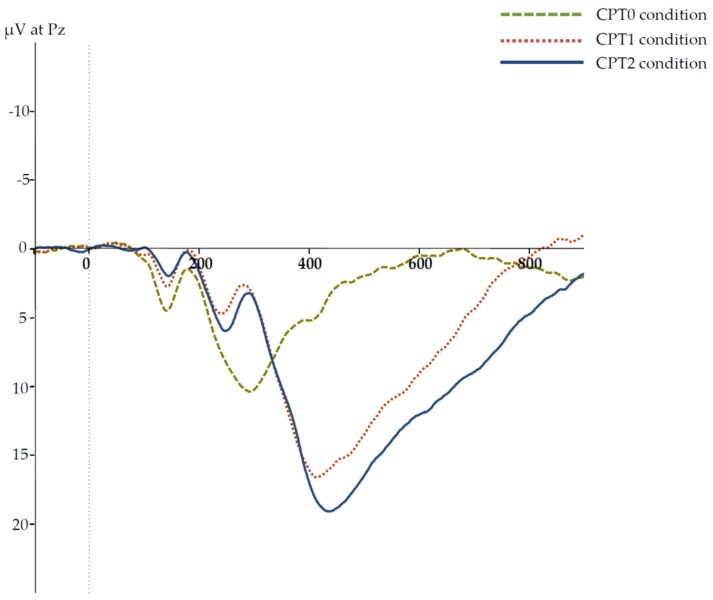
Grand average waves for the event-related potentials (ERPs) in the three CPT task conditions at the PZ electrode site. The zero point of the time scale refers to the onset. Negative is plotted upwards.

Grand averages of the ERPs in the three CPT conditions are presented in Figure 1. The respective descriptive statistics for the P3 amplitudes and latencies are given in Table 3. To examine differences between P3 latencies in the three CPT conditions for significance, a one-way ANOVA was computed, with P3 latencies in the three CPT conditions as three levels of a repeated-measures factor. Due to a violation of sphericity, the Greenhouse–Geisser correction was used with *ε* = 0.740. The main effect yielded statistical significance, *F*(1.480, 161.269) = 122.794, *p* < 0.001, *η_p_*² = 0.530. Planned comparisons revealed significantly shorter latencies in the CPT0 than in the CPT1 condition, *t*(109) = 10.385, *p* < 0.001, *d* = 0.990, as well as shorter latencies in the CPT1 than in the CPT2 condition, *t*(109) = 5.493, *p* < 0.001, *d* = 0.524.

The same analysis was computed for the P3 amplitude as a dependent variable to probe differences between P3 amplitude in the three CPT conditions. Again, sphericity was violated, so that the Greenhouse–Geisser adjustment was used with *ε* = 0.927. The main effect on the P3 amplitude was significant, *F*(1.854,202.091) = 185.224, *p* < 0.001, *η_p_*² = 0.630. Planned comparisons revealed that P3 amplitude was significantly larger in the CPT1 than in the CPT0 condition, *t*(109) = 14.741, *p* < 0.001, *d* = 1.405, as well as in the CPT2 than in the CPT1 condition, *t*(109) = 3.786, *p* < 0.001, *d* = 0.361. Thus, P3 amplitudes and latencies were sensitive to the experimental manipulation and increased monotonically from the CPT0 to the CPT2 condition.

Correlations between MA, P3 latencies, and P3 amplitudes are presented in Table 2. P3 amplitudes were not associated with MA in the three CPT conditions. The same was true for the P3 latency in the least demanding task condition. In both more demanding task conditions, P3 latency was negatively and significantly related to MA. 

In a next step, we submitted the P3 latencies from the three task conditions to stepwise regression analyses to predict MA. In the first model with P3 latency in the CPT0 condition as a predictor of MA, neither the *β* coefficient, *β* = −0.073, *p* = 0.446, nor the amount of explained variance, adjusted *R*^2^ = 0.000, were statistically significant. The second model with P3 latencies from the CPT0 and the CPT1 condition as predictors of MA led to a higher amount of explained variance, adjusted *R*^2^ = 0.043, with the *R*^2^ change being significant, *F*(1,107) = 6.234, *p* = 0.014. The *β* coefficient of P3 latency in the CPT1, *β* = −0.234, *p* = 0.014, but not the *β* coefficient of P3 latency in the CPT0 condition, *β* = −0.067, *p* = 0.479, yielded statistical significance. Comparing these first two models, P3 latency in the CPT1 condition explained a significantly larger portion of variance in MA compared to P3 latency in the CPT0 condition. Finally, with the third model, a statistically significant additional increase in the amount of explained variance was obtained (adjusted *R*^2^ = 0.101) compared to the second model, *F*(1,106) = 7.994, *p* = 0.006. Only the *β* coefficient of the P3 latency in the CPT2 condition, *β* = −0.297, *p* = 0.006, but not the *β* coefficients of P3 latency in the CPT1, *β* = −0.085, *p* = 0.421, and in the CPT0 condition, *β* = −0.085, *p* = 0.355, were statistically significant.

Also given in Table 2 are correlations among RT, P3 latency, and P3 amplitude. P3 latencies in the CPT1 and in the CPT2 conditions were significantly correlated with each other but not with P3 latency in the CPT0 condition. This result indicated a functional difference of P3 latency in the CPT0 compared to the CPT1 and CPT2 conditions. P3 latencies in the three CPT conditions were positively correlated with RTs, but only four out of the nine correlation coefficients yielded statistical significance. Correlations between P3 latencies and P3 amplitudes were all negative, but only four out of nine coefficients reached statistical significance. Finally, the P3 amplitudes in the three CPT conditions correlated significantly positively with each other, but negatively with RTs, except for the correlation between the P3 amplitude in the CPT0 condition and RT in the CPT2 condition (see Table 2). 

## 4. Discussion

The present study investigated the functional association between MA and the target-related P3 latency in the standard CPT as well as in two further conditions with variations of task demands. While in the standard condition (CPT1), a target (‘X’) had to be identified within a sequence of distractor stimuli, the process of selective identification was impeded in the attention-enhanced condition (CPT2) and omitted in the control condition (CPT0). This variation in task demands led to changes in P3 latency: The P3 latency was shorter in the CPT0 condition and longer in the CPT2 condition compared to the standard CPT condition. Concurrently, the correlational relationship between MA and P3 latency observed in the standard CPT condition ceased in the CPT0 condition but was even stronger in the CPT2 than in the CPT1 condition. As indicated by regression analyses, the significant amount of variance in MA explained by P3 latency in the standard CPT condition was also part of the variance explained by P3 latency in the attention-enhanced CPT2 condition. Even more importantly, the P3 latency in the attention-enhanced CPT2 condition explained an additional amount of variance of MA beyond and above the amount explained by the P3 latency in the standard CPT condition. This pattern of results strongly suggests that the functional correlation between MA and P3 latency depended on selective-attention demands.

In the standard CPT condition, attention needed to be directed to each stimulus to decide whether a target or a distractor was presented. In the CPT0 condition, only simple reactions were required so that the demands on selective attention were minimal. Finally, in the CPT2 condition, more selective attention than in the CPT1 condition was required to identify the target not only by its characteristic of being an ‘X’ but also by the additional feature of being italicized. Increasing RT as well as increasing P3 latencies and amplitudes from the CPT0 to the CPT2 condition indicated increasing selective-attention demands across CPT conditions.

In the standard CPT condition, the expected negative relationship between P3 latency and MA was observed, indicating that individuals with higher MA needed less time to correctly classify, and thus identify, the target stimulus than individuals with lower MA. This result was consistent with previous studies on the relation between MA and P3 latency using the oddball paradigm [25,29,30,31,32,33]. As outlined above, the main difference between the CPT, as used in the present study, and the oddball task was the composition of distractor stimuli. While only one distractor stimulus was used (and frequently presented) in the oddball task, different distractor stimuli were presented in the present standard CPT. In both tasks, however, the target stimulus was infrequently presented to be identified within a series of sequentially presented distractors. The negative relation between MA and P3 latency in the standard CPT condition supports the idea that the speed of identifying targets among distractors accounts for the relation between P3 latency and MA—as it was found for the many applications of the oddball task.

Consistent with the rationale of the present study, the absence of distractors made the identification of the target vs. nontarget stimulus unnecessary. Without this demand on selective attention, however, the latency of the P3 component was no longer related to MA. The absence of an association between MA and P3 latency in the CPT0 condition (i.e., a condition without selective-attention demands) was not unexpected given the results by McGarry-Roberts et al. [22] or Troche et al. [27]. In both studies, similar results were reported for the association between MA and P3 latencies in simple reaction time tasks as in the CPT0 condition of the present study.

P3 amplitude and latency as well as RT and error rates increased from the standard CPT1 to the attention-enhanced CPT2 condition indicating that the CPT2 condition required more selective attention to identify the italic ‘*X*’ among distractors (including the regular ‘X’). In line with our expectations, the functional relationship between MA and P3 latency became stronger in the CPT2 compared to the CPT1 condition. As indicated by regression analyses, P3 latency in the CPT2 condition not only explained an amount of variance in MA overlapping with P3 latency in the CPT1 condition but also a significant additional portion of variance in MA. This outcome provided further evidence for the notion that the selective-attention demands on target identification are decisive for the relation between MA and P3 latency.

Both RT and P3 latency proved to be similarly sensitive to our experimental manipulation. Nevertheless, the correlation between RT and P3 latency as two distinct speed measures was only weak and not significant in the CPT2 condition. These results provided additional converging evidence for the notion that RT and P3 latency represent functionally different processes [22]. Despite this apparent functional independence of P3 latency and RT, it was the most surprising result of the present study that RT was not (negatively) related to MA in any of the three task conditions. Many previous studies using the CPT analyzed errors of omission and commission regarding their relation to MA or as possible indicators of impaired attentional processes in neurologically ill individuals [38,39,40]. In the present study with healthy participants, both kinds of errors were extremely rare and no correlations between intelligence and errors of omission or commission could be obtained. Since errors did not differ between individuals with higher and those with lower MA and the use of different cognitive strategies was unlikely, the standard CPT met the preconditions for a systematic relationship between RT and MA [1,2]. It should be noted, however, that a lacking negative relation between MA and RT is not unusual in the field of mental chronometry [41,42]—even though the majority of studies revealed a weak to modest relationship between MA and RT [3].

## 5. Conclusions

Previous research on the relation between MA and P3 latency has been rather inconclusive reporting negative correlations [25,29,30,33], positive correlations [28] or no significant correlations at all [22,27]. The present study provided first evidence for the notion that task demands on selective attention play a crucial role for the expected negative functional relationship between MA and P3 latency, as proposed within the conceptual framework of the mental speed approach. The negative relation between MA and P3 latency increased systematically with an increase of selective attention required by the task used to elicit the P3 component. As P3 latency and RT were functionally independent from each other, the failure to obtain a relationship between RT and MA did not necessarily hamper the interpretation of the relation between MA and P3 latency.

## Figures and Tables

**Table 1:** Descriptive statistics of reaction times, errors of commission, and errors of omission in the three conditions of the continuous performance test (CPT) in 110 participants. No errors of commission were possible in the CPT0 condition.

Condition	Reaction Times (ms)				Errors of Commission (%)				Errors of Comission (%)			
	M	SD	Min	Max	M	SD	Min	Max	M	SD	Min	Max
CPT0	250	23	211	321	-	-	-	-	0.015	0.028	0	0.120
CPT1	416	41	327	530	0.005	0.007	0	0.031	0.003	0.011	0	0.080
CPT2	482	46	381	613	0.012	0.011	0	0.069	0.006	0.017	0	0.031

M = Means, SD = standard deviation, Min = minimum, Max = maximum.

**Table 2:** Pearson correlations between reaction times, P3 latencies, and P3 amplitudes in the three conditions of the continuous performance test (CPT0 to CPT2), as well as scores on the CFT-20 R in 110 participants.

Variable	Task/Condition	Reaction Times			P3 Latencies			P3 Amplitudes		
		CPT0	CPT1	CPT2	CPT0	CPT1	CPT2	CPT0	CPT1	CPT2
	CFT total	–0.112	–0.002	0.081	–0.073	–0.236 *	–0.336 ***	0.072	0.187 *	0.135
Reaction times	CPT0		0.437 ***	0.366 ***	0.213 *	0.133	0.041	–0.244 **	–0.272 **	–0.249 **
	CPT1			0.600 ***	0.077	0.293 **	0.215 *	–0.226 **	–0.339 ***	–0.241 **
	CPT2				0.209 *	0.124	0.086	–0.144	–0.218 *	–0.305 **
P3 latencies	CPT0					0.029	–0.046	–0.262 **	–0.170 *	–0.273 **
	CPT1						0.500 ***	–0.010	–0.154	–0.160 *
	CPT2							0.111	–0.103	–0.016
P3 amplitudes	CPT0								0.404 ***	0.339 ***
	CPT1									0.686 ***

* *p* < 0.05; ** *p* < 0.01; *** *p <* 0.001 (one-tailed).

**Table 3:** Descriptive statistics of P3 latencies and P3 amplitudes in the three CPT conditions in 110 participants.

Condition	P3 Latencies (ms)				P3 Amplitudes (μV)			
	M	SD	Min	Max	M	SD	Min	Max
CPT0	371	39	300	543	7.78	3.73	0.69	26.01
CPT1	418	30	360	526	14.50	5.25	3.00	38.80
CPT2	438	29	361	535	16.49	5.87	6.59	42.44

M = Means, SD = standard deviation, Min = minimum, Max = maximum.

## Supplementary Materials

The following are available online at http://www.mdpi.com/2076-3425/9/2/28/s1, Figure S1: Grand averages (solid lines) and standard deviations (dotted lines) of the event-related potentials in the three CPT conditions; Figure S2: Grand average (solid line) and standard deviation (dotted line) of the event-related potentials in the CPT0 condition; Figure S3: Grand average (solid line) and standard deviation (dotted line) of the event-related potentials in the CPT1 condition; Figure S4: Grand average (solid line) and standard deviation (dotted line) of the event-related potentials in the CPT2 condition.
