# Supplementary material for: On the Relationship between P3 Latency and Mental Ability as a Function of Increasing Demands in a Selective Attention Task
Source: Brain Sci. 2019 Jan 29;9(2):28. doi: 10.3390/brainsci9020028 (PMC6406371; doi:10.3390/brainsci9020028)
Supplement: Supplementary file 1 [file brainsci-09-00028-s001.pdf]

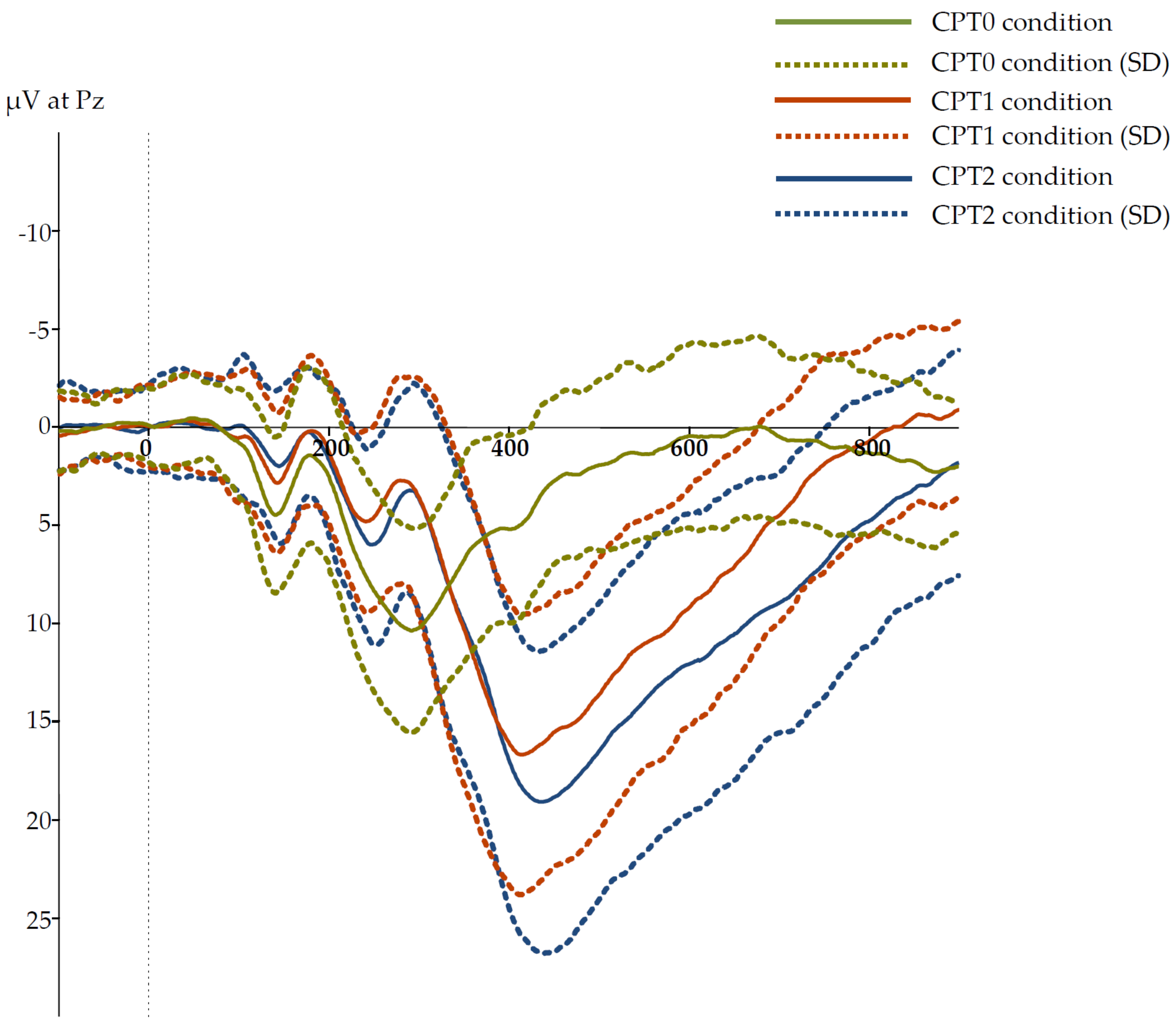

Figure S1. Grand averages (solid lines) and standard deviations (dotted lines) of the event-related potentials in the three CPT conditions.

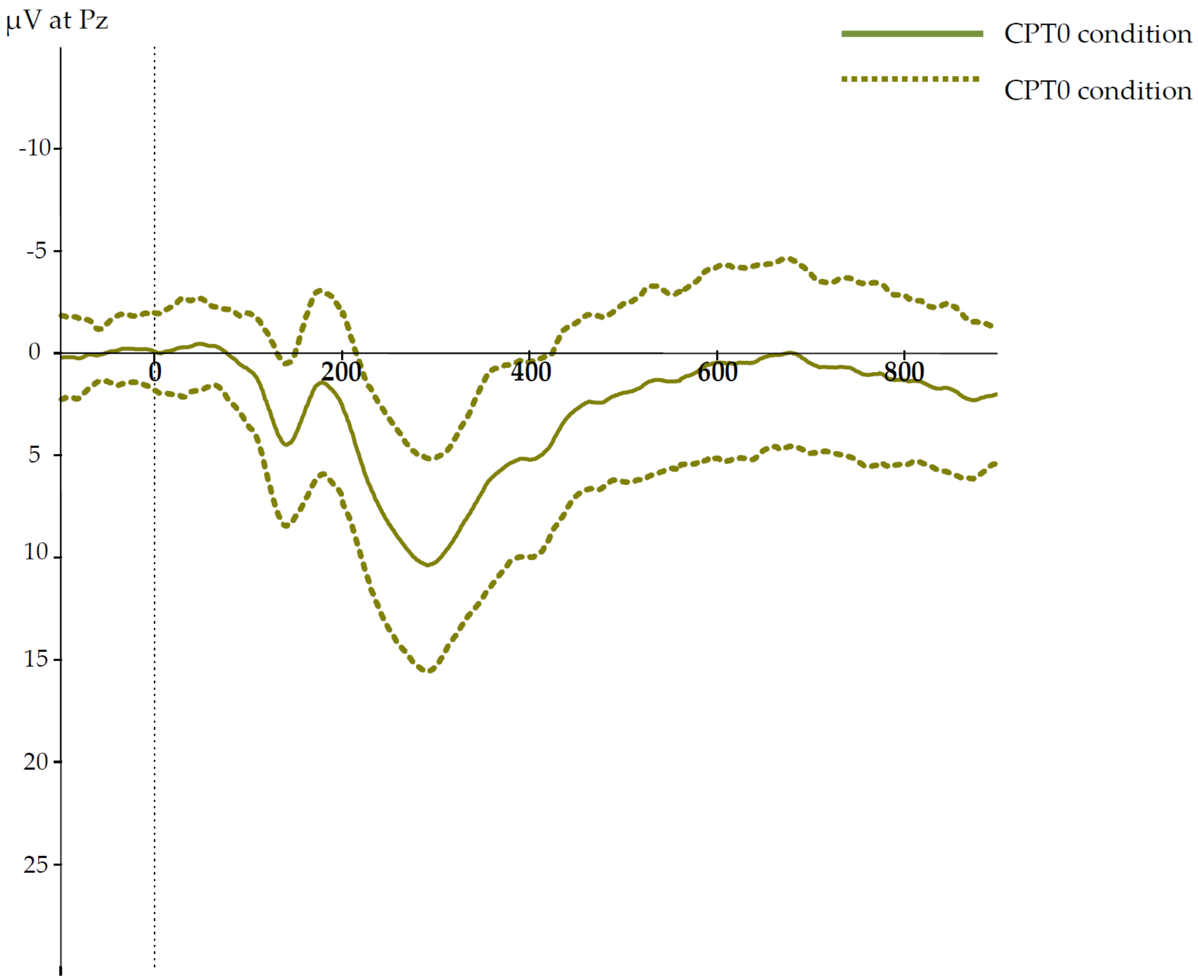

Figure S2. Grand average (solid line) and standard deviation (dotted line) of the event-related potentials in the CPT0 condition.

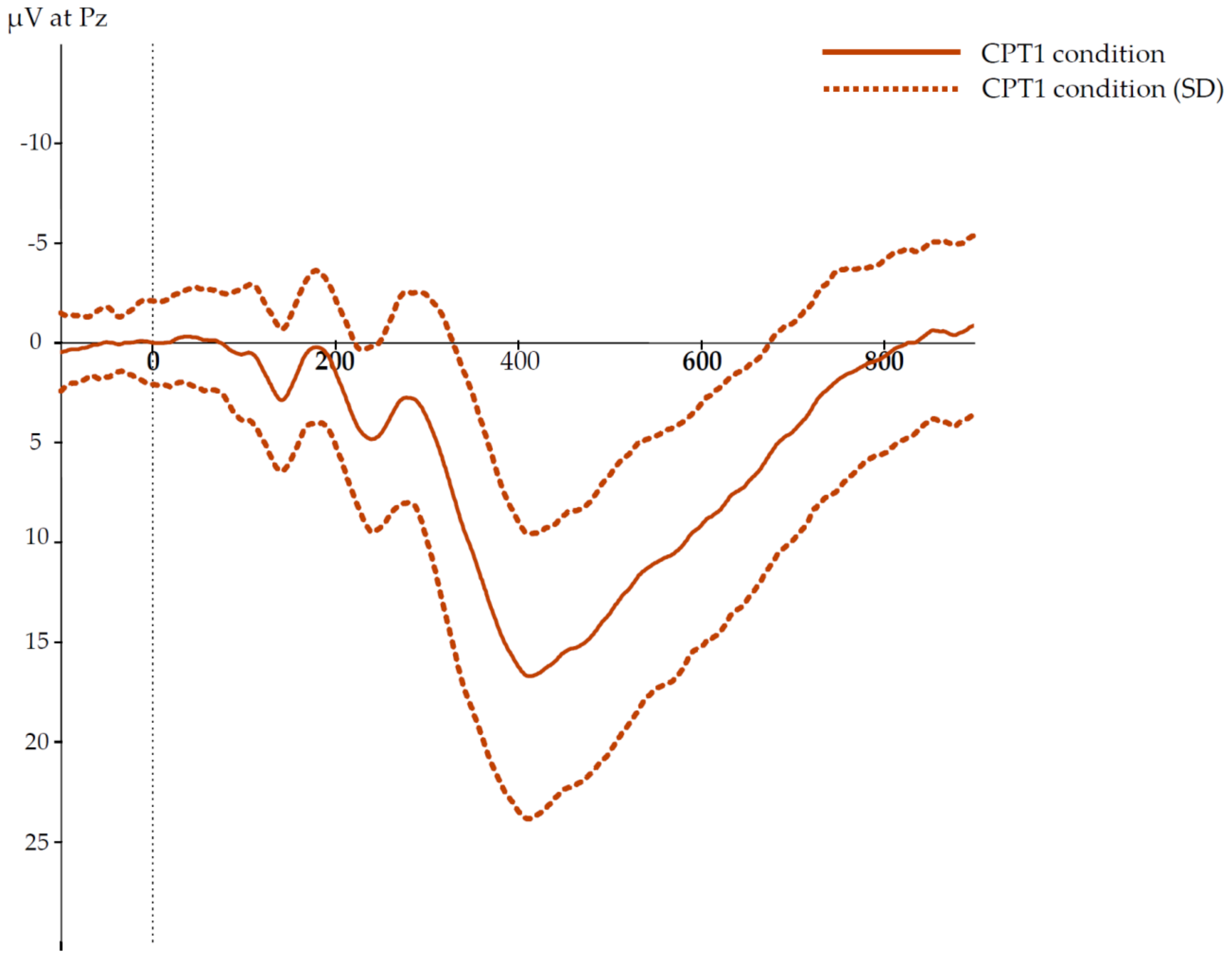

Figure S3. Grand average (solid line) and standard deviation (dotted line) of the event-related potentials in the CPT1 condition.

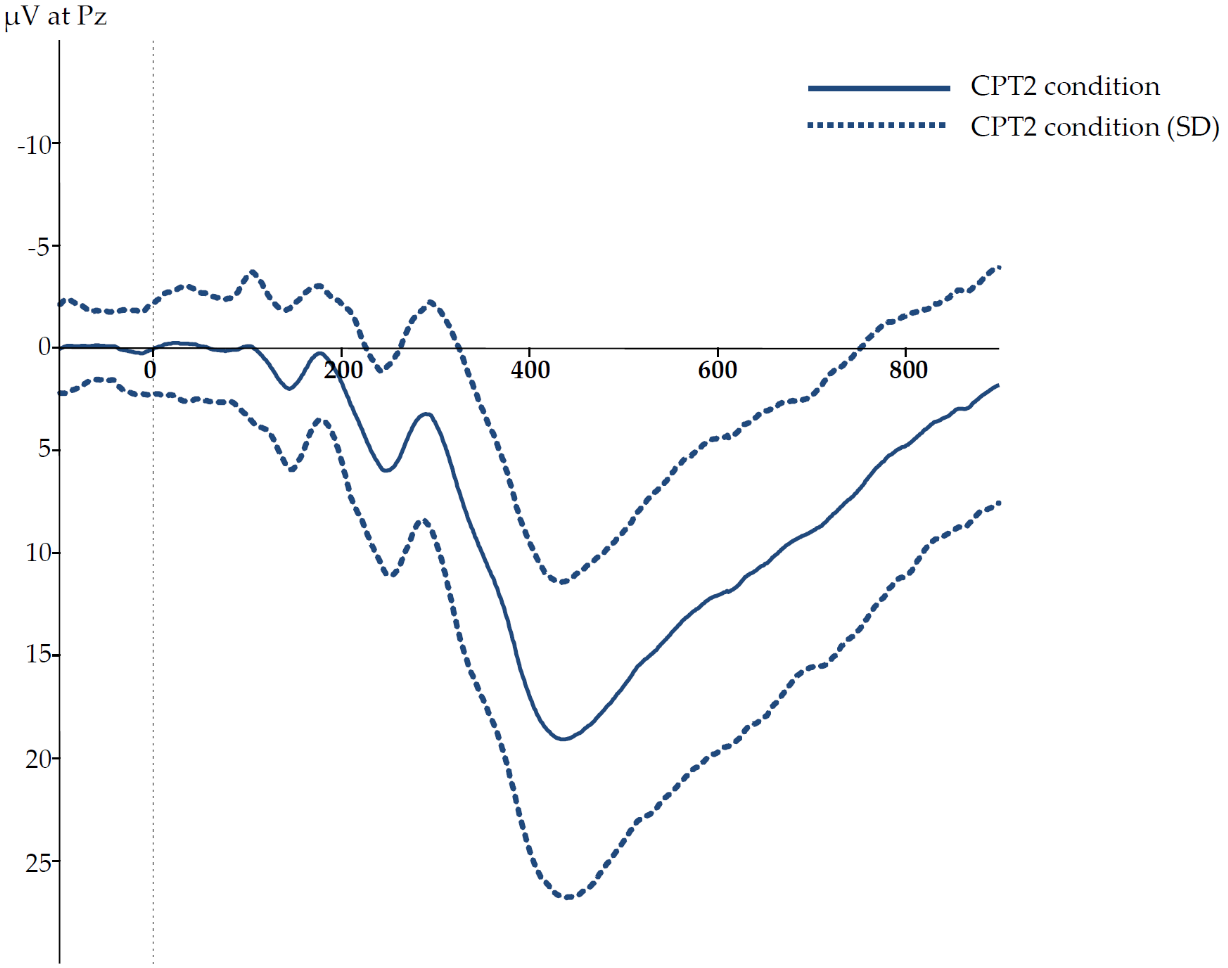

Figure S4. Grand average (solid line) and standard deviation (dotted line) of the event-related potentials in the CPT2 condition.
